# Supplementary material for: Antiviral Evaluation of New Synthetic Bioconjugates Based on GA-Hecate: A New Class of Antivirals Targeting Different Steps of Zika Virus Replication
Source: Molecules. 2023 Jun 21;28(13):4884. doi: 10.3390/molecules28134884 (PMC10343505; doi:10.3390/molecules28134884)
Supplement: Supplementary file 1 [file molecules-28-04884-s001.zip › molecules-2432352-supplementary.pdf]

Supplementary Material

Antiviral Evaluation of New Synthetic Bioconjugates Based on GA-Hecate: A New Class of Antivirals Targeting Different Steps of Zika Virus Replication

Paulo Ricardo da Silva Sanches [1,2,3,*], Ricardo Sanchez-Velazquez [2], Mariana Nogueira Batista [4], Bruno Moreira Carneiro [5], Cintia Bittar [5], Giuditta De Lorenzo [2], Paula Rahal [6], Arvind H. Patel [2], Eduardo Maffud Cilli [3,*]

[1] School of Pharmaceutical Science, São Paulo State University, Araraquara 14800-903 SP, Brazil
[2] MRC—University of Glasgow Centre for Virus Research, University of Glasgow, G12 8QQ Glasgow, UK; ricardo.sanchez@biontech.de (R.S.-V.); giuditta.delorenzo@glasgow.ac.uk (G.D.L.); arvind.patel@glasgow.ac.uk (A.H.P.)
[3] Institute of Chemistry, São Paulo State University, Araraquara 14800-900, SP, Brazil
[4] Laboratory of Virology and Infectious Diseases, The Rockefeller University, New York, NY 10065, USA; mnogueirab@rockefeller.edu (M.N.B.); cintia.bittar@unesp.br (C.B.)
[5] School of Health Science, Federal University of Rondonópolis, Rondonópolis 78736-900, MT, Brazil; bruno@ufr.edu.br
[6] Institute of Bioscience, Humanities and Exact Science, São Paulo State University, S.J. Rio Preto 15054-000, SP, Brazil; p.rahal@unesp.br
* Correspondence: paulo.sanches@unesp.br (P.R.d.S.S.); eduardo.cilli@unesp.br (E.M.C.)

1. Introduction

Here, we show the mass spectra of Hecate peptide after incubation with human blood serum and chromatograms and the mass spectra of the synthetic peptides (GA-Hecate and GA-Metabolites). Also, we describe the analyses used to identify characteristics from different ZIKV strains and the selection criteria applied to choose PE243 and MP1751 strains in this study.

2. Materials and Methods

Mass spectrometry. Compounds solutions were analyzed by mass spectrometry using a Bruker spectrometer in electrospray positive mode with direct injection. Spectra were analysed using Spectra Analysis Software using the relationship between molecular weight (m) and charge (z) (m/z). Fragments of Hecate were identified, and the amino acid sequence was determined by m/z comparison.

Peptide synthesis and purification. Peptide synthesis was performed via automated solid-phase using the standard Fmoc (9-fluorenylmethyloxycarbonyl) protocol in a TRIBUTE-UV (Protein) synthesizer on a Rink-MBHA resin (Hecate, GA-Hecate) and Wang resin (GA-Metabolites). Compounds were purified by high-performance liquid chromatography (HPLC) and the identity was confirmed by electrospray mass spectrometry.

ZIKV strains genome and protein E comparison. Complete genome and protein sequences of different Zika virus strains were obtained from GenBank and compared using the BLAST software. Images were constructed using data reported under PDB accession numbers 3JZ7 for M-E protein and 5JHM for E-dimer. Peptides containing fragments 121-180 (N-acetyl-D-glucosamine region) were modeled using Swiss Model software and the PDBs were analyzed by PyMol software[1,2].

3. Results and Discussion

3.1. Hecate incubation with Human Serum

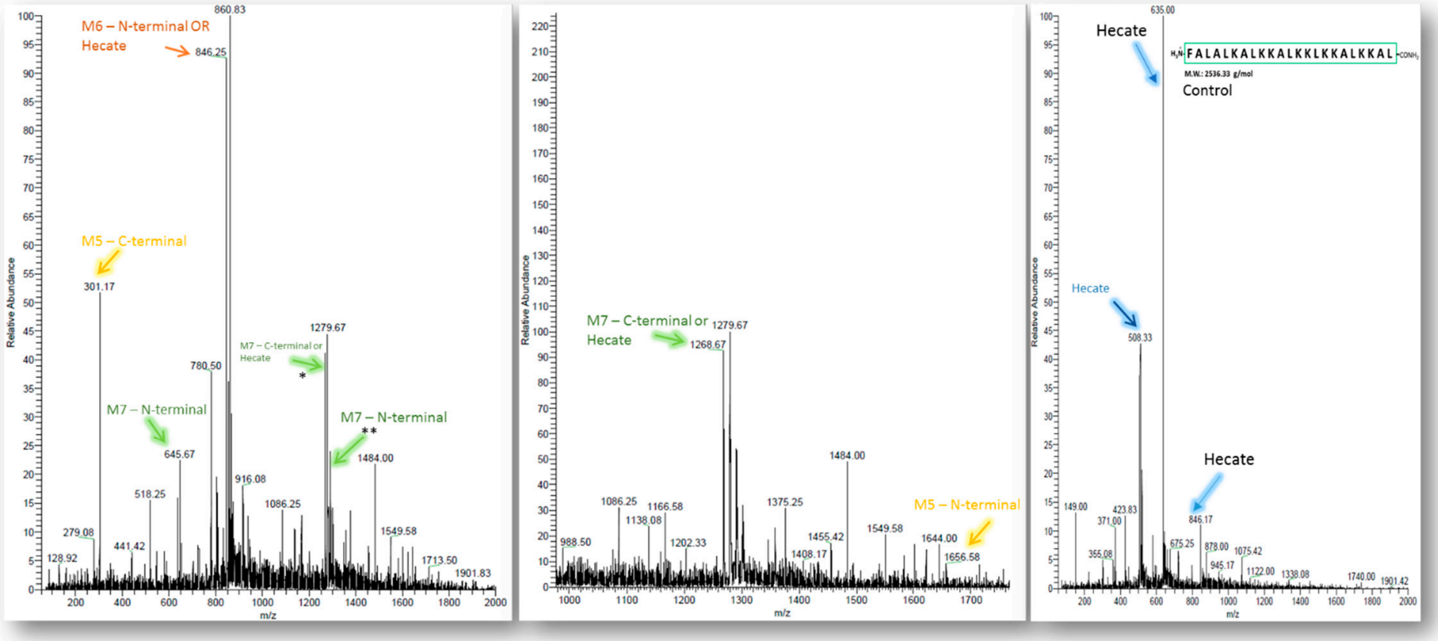

Figure S1. Identification of Hecate peptide fragments after incubation of GA-Hecate with human blood serum. Mass spectra show the relationship m/z and the respective metabolites are indicated by the arrows. *m/z: 1268.67 g/mol; **1285.45 g/mol.

3.2. Peptide synthesis and purification

The GA-peptides were characterized after purification by high-performance liquid chromatography (HPLC – Supplementary figure 2). The m/z (g/mol) of GA-Hecate (+3: 896.59; +4: 672.92; +5: 538.44 and +6: 448.77), GA-Metabolite 5 (+2: 905.01; +3: 603.68 and +4: 452.98), GA-Metabolite 6 (+1: 998.58; +2: 499.63) and GA-Metabolite 7 (+2: 719.80 and +3: 480.25) confirmed that the synthesis was successful (Supplementary Figure 3 (a), (b), (c), and (d), respectively).

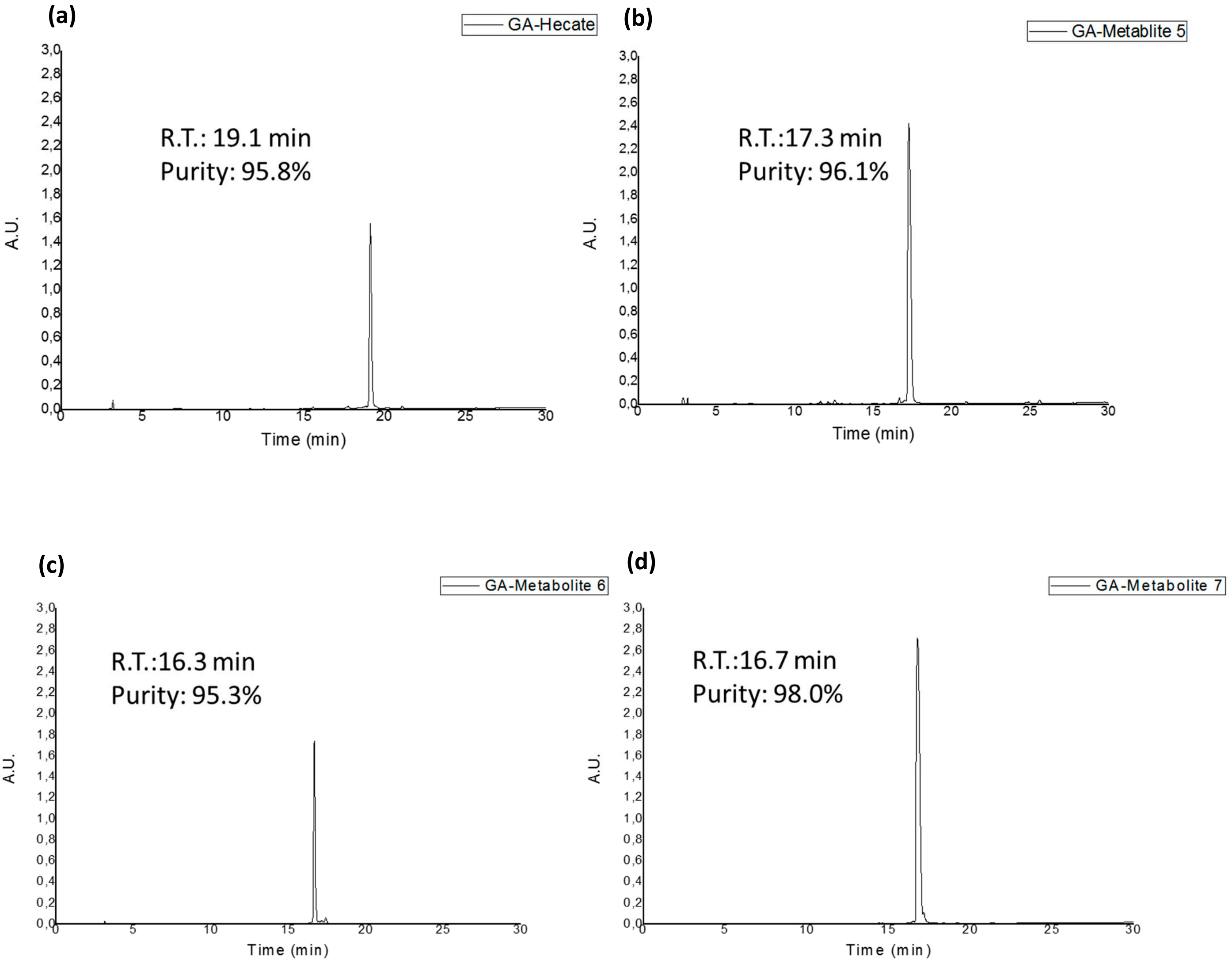

Figure S2. Chromatograms of pure peptides. **(a)** GA-Hecate **(b)** GA-Metabolite 5 **(c)** GA-Metabolite 6 and **(d)** GA-Metabolite 7. R.T.: Retention Time. The purities (%) were calculated using integration methods for each chromatogram comparing the area percent of the main peak and the impurities.

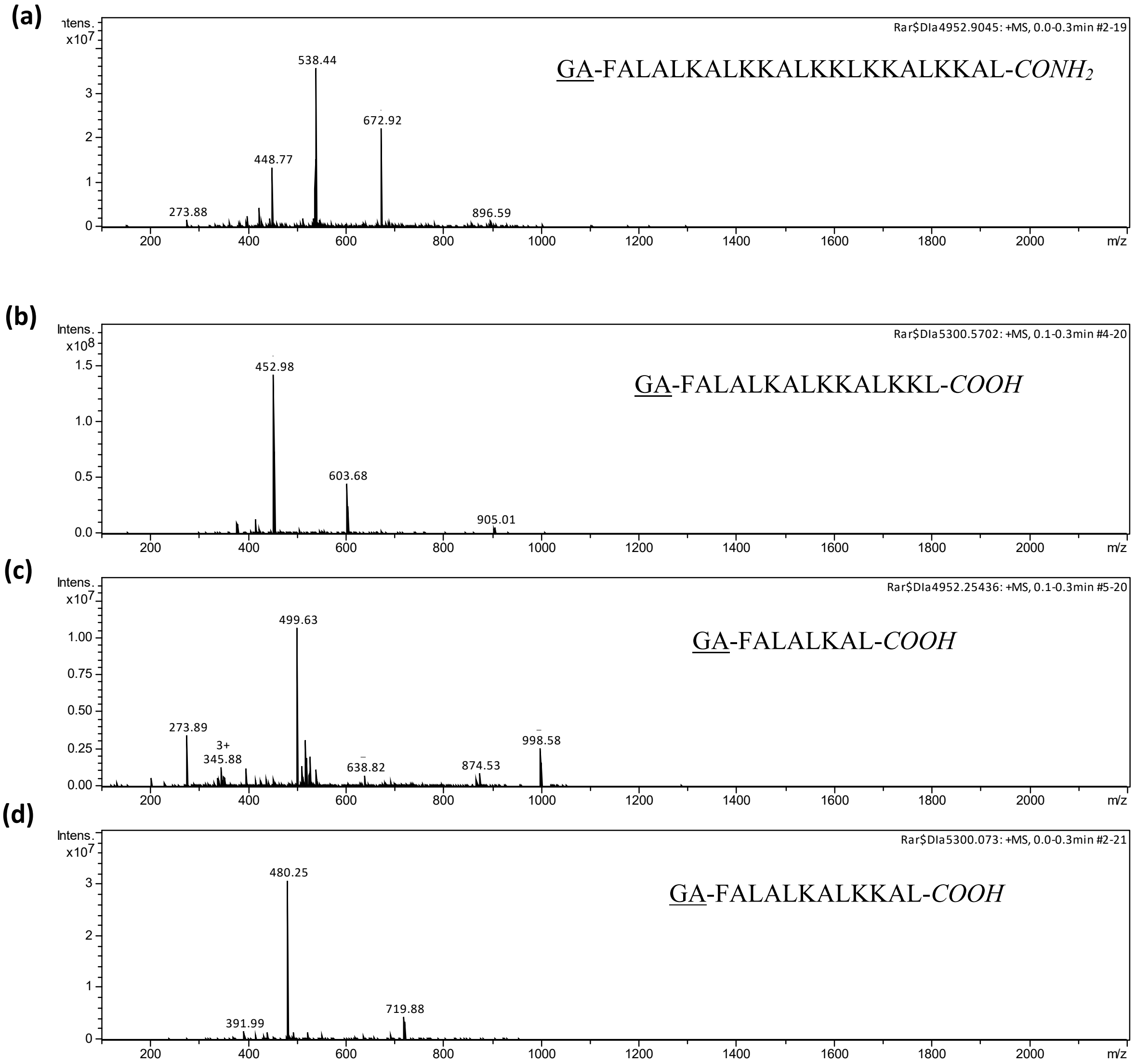

Figure S3. Characterization of peptides by mass spectrometry. **(a)** GA-Hecate (b) GA-Metabolite 5 **(c)** GA-Metabolite 6 and **(d)** GA-Metabolite 7.

3.3. ZIKV strains genome and protein E comparison

Several phylogenetic studies have shown that there are differences in amino acid sequences between historical ZIKV strains (Africa) and recent outbreak isolated strains (Asia)[3–5]. Many differences in these amino acids have been implicated in transmission, immunity, infectivity, tropism, and disease enhancement[3,4]. Using bioinformatics, we compared the genome of different ZIKV (historical lineages from Africa and a recent America outbreak) and the E protein of the Brazilian ZIKV strain (PE243) and historical African ZIKV strains (MR766 and MP1751). Our results show that there are differences in the genome of all of the strains, but strains MR766 and MP1751 have shown 94.26% of similarity. Comparing the genome of African strains with America's outbreak isolated strains, less than 90% similarity has been found (Supplementary Table 1). The E amino acid sequences from three different ZIKV strains (PE243, MP1751, and MR766) were compared (Supplementary Table 1). Our results suggest a high amino acid similarity between these strains (98.41% of identity between African strains, 96.00% between PE243 and MR766, and 97.82 between PE243 and MP1751

(Supplementary Table 2). However, MR766 (AF) compared with MP1751 (AF) and PE243 (BR) doesn't present "sequon" sequence (Asn-Xaa-Thr/Ser), which has been reported as an important binding glycan site[6–8]. PE243 and MP1751 present ^{154}VNDT^{157} sequence while MR766 doesn't present those amino acids. A comparison of the tertiary structure of peptides containing the amino acid sequence 121-180 shows that this region contains anti-parallel sheets in all strains (Supplementary figure 3). Also, all strains contain alpha-helix structures, but in different regions. MP1751 and PE243 form a helix with amino acids ^{154}VNDTGHETD^{162} (glycan loop) while MR766 forms a helix with ^{147}QHSGMI^{152}. Additionally, Tyr^{158} presents in MR766 has been replaced by His^{158} in PE243 and MP1751 strains, which could explain the flexibility of this region in MR766 strain arising due to decreasing interaction between the side chain of amino acids (histidine side chain has five atoms ring with two nitrogen atoms while tyrosine side chain has benzene ring) and also because of the steric hindrance.

Table S1. Genome comparison of different ZIKV strains.

**Strain 1**	**Strain 2**	**Genome Identity (%)**
MP1751 (KY288905.1) ***(AF)***	PE243 (MF352141.1) ***(BR)***	89.12
MP1751 (KY288905.1) ***(AF)***	MR766 (KX377335.1) ***(AF)***	94.26
PE243 (MF352141.1) ***(BR)***	MR766 (KX377335.1) ***(BR)***	89.03
PE243 (MF352141.1) ***(BR)***	ZIKV_isolate Brazil_2015_MG (KX811222.1) ***(BR)***	99.71
PE243 (MF352141.1) ***(BR)***	ZIKV_isolate Rio_U1 (KU926309.2) ***(BR)***	99.71
PE243 (MF352141.1) ***(BR)***	ZIKV_isolate Paraiba_01 (KX811222.1) ***(BR)***	99.76
PE243 (MF352141.1) ***(BR)***	ZIKV/H.sapiens/Brazil/Natal/2015 (NC_035889.1) ***(BR)***	99.69
PE243 (MF352141.1) ***(BR)***	ZIKV isolate Haiti/0033/2014 (KY415987.1) ***(HAI)***	99.67
PE243 (MF352141.1) ***(BR)***	ZIKV isolate PRVABC59 (MH158237.1) ***(PR)***	99.69
MP1751 (KY288905.1) ***(AF)***	ZIKV isolate Haiti/0033/2014 (KY415987.1) ***(HAI)***	89.22
MP1751 (KY288905.1) ***(AF)***	ZIKV isolate PRVABC59 (MH158237.1) ***(PR)***	89.11

BR: Brazil; AF: Africa; HAI: Haiti; PR: Puerto Rico.

Code into parentheses means GenBank code access.

Table S2. Amino acid sequence comparison of PE243, MP1751, and MR766 Zika virus strains.

Strain 1	Strain 2	Amino acid Identity (%)
PE243	MP1751	97.82
PE243	MR766	96.00
MP1751	MR766	98.41

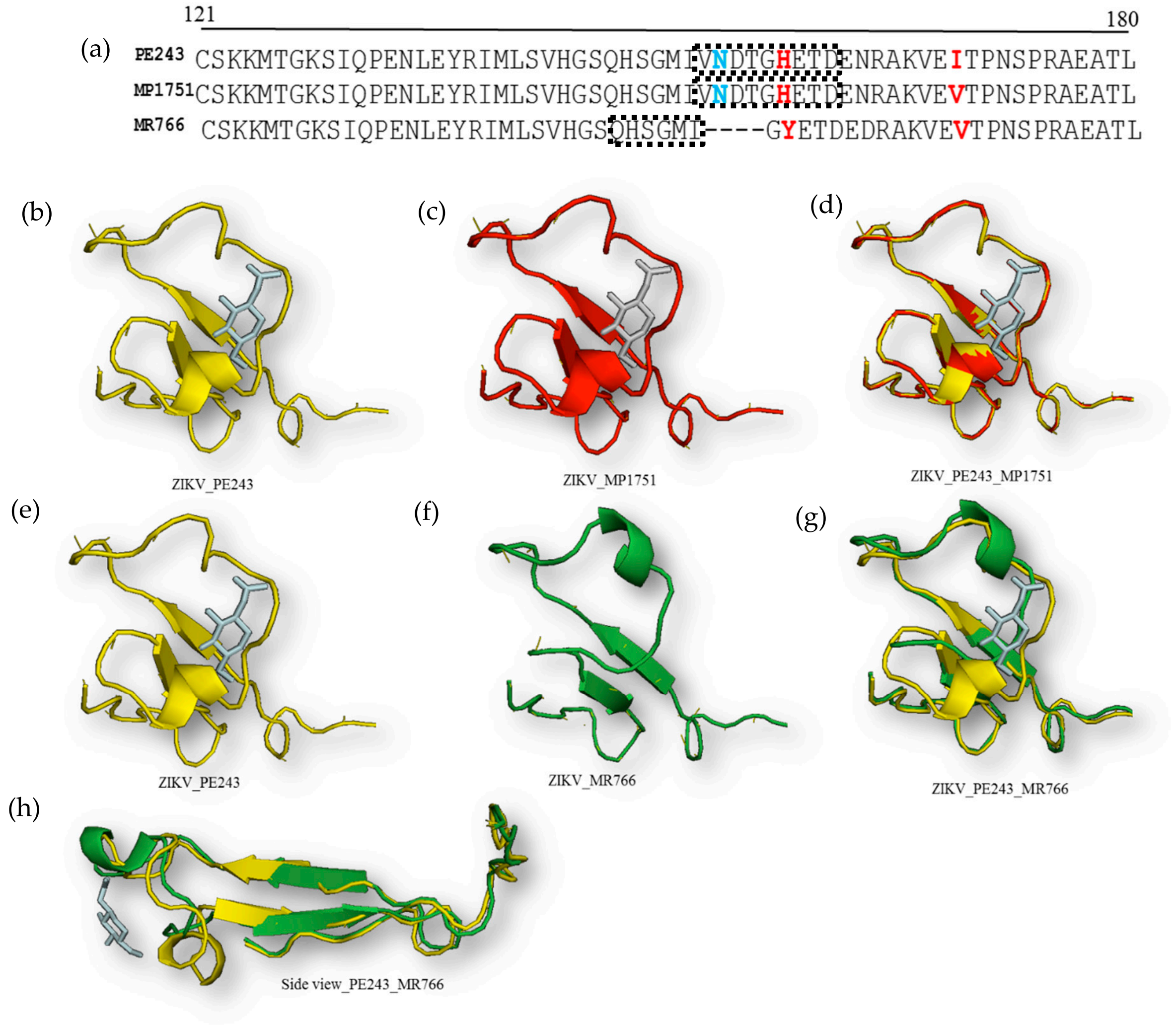

Figure S4. Peptide 121-180 from protein E. Comparison of three ZIKV strains PE243, MP1751, and MR766. **(a)** Peptide amino acid sequence comparison. Regions highlighted with dotted lines are showing amino acids in α-helix structure **(b)** Peptide structure from PE243 strain (yellow) **(c)** Peptide structure from MP1751 strain (red) **(d)** Comparison structures between PE243 and MP1751 strains **(e)** Peptide structure from PE243 strain **(f)** Peptide structure from MR766 strain (green) **(g)** Comparison structures between PE243 and MR766 strains **(h)** Peptide side view from PE243 (yellow) and MR766 (green).

Conclusion

- We identified a series of metabolites from Hecate peptide after human blood serum incubation by mass spectrometry, synthesized and conjugated them with gallic acid. All synthetic GA-peptides were generated with high levels of purity (> 95.0%).

- Despite the protein E amino acid sequence similarities between MP1751 and PE243 and because the mainstream of strains isolated during the outbreak presented high similarity with those strains, we decided to test the synthetic compound against both.

References

1. Schwede, T.; Kopp, J.; Guex, N.; Peitsch, M.C. SWISS-MODEL: An Automated Protein Homology-Modeling Server. *Nucleic Acids Res.* **2003**, *31*, 3381–3385, doi:10.1093/nar/gkg520.
2. Yuan, S.; Chan, H.C.S.; Hu, Z. Using PyMOL as a Platform for Computational Drug Design. *Wiley Interdiscip. Rev. Comput. Mol. Sci.* **2017**, *7*, doi:10.1002/wcms.1298.
3. Simonin, Y.; Loustalot, F.; Desmetz, C.; Foulongne, V.; Constant, O.; Fournier-Wirth, C.; Leon, F.; Molès, J.P.; Goubaud, A.; Lemaitre, J.M.; et al. Zika Virus Strains Potentially Display Different Infectious Profiles in Human Neural Cells. *EBioMedicine* **2016**, *12*, 161–169, doi:10.1016/j.ebiom.2016.09.020.
4. Beaver, J.T.; Lelutiu, N.; Habib, R.; Skountzou, I. Evolution of Two Major Zika Virus Lineages: Implications for Pathology, Immune Response, and Vaccine Development. *Front. Immunol.* **2018**, *9*, doi:10.3389/fimmu.2018.01640.
5. Liu, Z.Y.; Shi, W.F.; Qin, C.F. The Evolution of Zika Virus from Asia to the Americas. *Nat. Rev. Microbiol.* **2019**, *17*, 131–139, doi:10.1038/s41579-018-0134-9.
6. Kasturi, L.; Chen, H.; Shakin-Eshleman, S.H. Regulation of N-Linked Core Glycosylation: Use of a Site-Directed Mutagenesis Approach to Identify Asn-Xaa-Ser/Thr Sequons That Are Poor Oligosaccharide Acceptors. *Biochem. J.* **1997**, *323*, 415–419, doi:10.1042/bj3230415.
7. Routhu, N.K.; Lehoux, S.D.; Rouse, E.A.; Bidokhti, M.R.M.; Giron, L.B.; Anzurez, A.; Reid, S.P.; Abdel-Mohsen, M.; Cummings, R.D.; Byrareddy, S.N. Glycosylation of Zika Virus Is Important in Host–Virus Interaction and Pathogenic Potential. *Int. J. Mol. Sci.* **2019**, *20*, doi:10.3390/ijms20205206.
8. Nilsson, I.M.; Von Heijne, G. Glycosylation Efficiency of Asn-Xaa-Thr Sequons Depends Both on the Distance from the C Terminus and on the Presence of a Downstream Transmembrane Segment. *J. Biol. Chem.* **2000**, *275*, 17338–17343, doi:10.1074/jbc.M002317200.
